# Meta-analysis of over 8,000 individuals from Hawai‘i and Samoa for genetic associations to cardiometabolic phenotypes

**DOI:** 10.1371/journal.pgen.1012342

**Published:** 2026-10-21

**Authors:** Bryan L. Dinh, Xinran Wang, Xin Sheng, Peggy Wan, Amit Kumar Srivastava, Take Naseri, Satupa‘itea Viali, The Samoan Obesity, Lifestyle and Genetic Adaptations (OLaGA) Study Group, Lynne R. Wilkens, Loïc Le Marchand, Christopher A. Haiman, Daniel E. Weeks, Charleston W. K. Chiang, Jenna C. Carlson

**Affiliations:** 1 Center for Genetic Epidemiology, Department of Population and Public Health Sciences, University of Southern California, Los Angeles, California, United States of America; 2 Department of Quantitative and Computational Biology, University of Southern California, Los Angeles, California, United States of America; 3 Department of Human Genetics, University of Pittsburgh, Pittsburgh, Pennsylvania, United States of America; 4 Department of Biostatistics and Health Data Science, University of Pittsburgh, Pittsburgh, Pennsylvania, United States of America; 5 Ministry of Health, Apia, Samoa; 6 Department of Epidemiology, Brown University, Providence, Rhode Island, United States of America; 7 Oceania University of Medicine, Apia, Samoa; 8 Department of Chronic Disease Epidemiology, Yale School of Public Health, New Haven, Connecticut, United States of America; 9 Population Sciences in the Pacific Program, Cancer Center, University of Hawaiʻi, Honolulu, Hawai‘i, United States of America; The University of Texas MD Anderson Cancer Center, UNITED STATES OF AMERICA

## Abstract

Although genome-wide association studies (GWAS) now routinely reveal genetic associations and biological insights in millions of individuals, underrepresentation of global populations, such as those from Polynesia, continue to persist. These exclusions, often driven by logistical challenges and lack of data, prevent systematic identification of population-enriched associations, such as the association of the missense variant at the CREBRF locus to BMI and type 2 diabetes discovered commonly occurring in Polynesian populations due to its rarity in global populations. Armed with the recently updated TOPMed imputation panel that could benefit studies in diverse populations that previously had poorer imputation performance, we performed the first GWAS of Native Hawaiians and Samoans, the largest to date of Polynesian-ancestry populations (combined N up to 8,461) to identify population-enriched associations for 13 adiposity and cardiometabolic traits available across both cohorts: BMI, fasting glucose, fasting insulin, HDL, height, hip circumference, HOMA-IR, LDL, T2D, total cholesterol, triglycerides, waist circumference, and waist-hip ratio. We found 25 trait-loci associations that met genome-wide significance: 20 previously reported or known associations and 5 putatively new associations via meta-analysis. In particular, with improved statistical power, we were able to confirm the previously suggestive association between the missense *CREBRF* variant with fasting glucose levels. The remaining 4 potentially novel loci-trait associations for BMI, LDL, and waist-hip ratio, however, were not replicated in All-of-Us. The lack of Polynesian-enriched findings outside of the *CREBRF* locus informs the bounds of the effect sizes or frequencies of any enriched variants and suggests that further expansion of cohort sizes from this region of the world and improved imputation references specific to these populations are needed to identify more population-enriched associations.

## Author summary

Understudied populations, like those from the Pacific Islands, are often excluded from large-scale human genetic studies due to small cohort sizes and unique study considerations, resulting in a growing disparity of benefits compared to well-studied groups. Although Polynesian-ancestry individuals remain underrepresented, recent updates to publicly available genomic resources such as one provided by the TOPMed initiative provide a renewed potential to map Polynesian-enriched variants for association with complex traits. We leveraged these resources to conduct the largest GWAS in Polynesian-ancestry individuals to date in up to 8,461 individuals, combining Native Hawaiian and Samoan cohorts for the first time. Across 13 adiposity and cardiometabolic traits, we identified 775 significant variant-trait associations at 25 loci, including five putative and 20 previously established loci-trait associations. Notably, we illustrated the effects of the Polynesian-enriched *CREBRF* missense variant on multiple traits but failed to replicate four other potentially novel associations for BMI, LDL, and waist-hip ratio. Our results help define the realistic bounds of associations disproportionately impacting these populations and underscore the need for further increased representation to identify more population-enriched genetic risk factors.

## Introduction

Native Hawaiians and Samoans are underserved populations with Polynesian ancestry that experience different incidence rates of BMI, type 2 diabetes (T2D), cancers, and cardiovascular disease (CVD) among others, compared to other continental populations [1–9]. As data are scarce and available cohorts are small, they are often excluded from investigations of the genetic or environmental underpinnings of complex traits and diseases. Additionally, the lack of adequate genomic resources (both in available microarray data and genotype imputation references) and complex population structure and admixture history have also complicated their inclusion in genetic investigations [7,10–12]. This presents a particular challenge for identifying associations driven by population-enriched alleles – alleles that are tens if not hundreds of folds more common in Polynesia compared to the rest of the world and contribute disproportionately to phenotypic variation and disease risk in Hawaiians, Samoans, and other Pacific Islanders [13–16]. As these variants are rare outside of Polynesia, they can only be discovered efficiently with cohorts derived from this region.

While both Native Hawaiians and Samoans represent Polynesian ancestries at the macro-geographical level, there are fine-scale differences between the two populations. Specifically, Native Hawaiians represent eastern Polynesian-related ancestries and, due to European colonization and admixture, at minimum additionally incorporate East Asian-, European-, and African-related ancestries [17]. By comparison, Samoans are less admixed and represent predominantly western Polynesian-related ancestries [18]. Nevertheless, the two populations as well as other Pacific Islander populations have a shared history and genetic architecture [19,20]. For instance, a Polynesian-enriched missense allele of the variant rs373863828 (chr5:173108771) in *CREBRF* was first discovered in a Samoan study and was common among Native Hawaiian and other Pacific Islander populations (~9–26%) with similar effect sizes on BMI [14,15,21–23]. This allele is virtually non-existent in other continental ancestries (frequency < 2x10^-4^ in gnomAD [24]). Some studies have also suggested that this *CREBRF* missense variant may be associated with other adiposity or metabolic phenotypes, though the statistical evidence is marginal due to lack of power [14,15]. Further, a multiple cohort study including Samoans and other individuals of Pacific Islander ancestry discovered another Polynesian-specific missense *CETP* variant, rs1597000001 (chr16:56971035), associated with High Density Lipoprotein Cholesterol (HDL-C) and Low Density Lipoprotein Cholesterol (LDL) levels [16]. Hence, combining both populations should improve statistical power to discover additional population-enriched associations shared across Polynesia.

More importantly, since the reporting of those studies, publicly available imputation panels such as the state-of-the-art TOPMed imputation server have improved genotype imputation accuracy through increases in the number of variants (>445 million), number of individuals (>133k), and diversity of representation [25,26], with benefits already observed for Latino and African American populations. In particular, the previously unimputable *CREBRF* variant [15] can now be imputed using TOPMed references to high accuracy (Rsq > 0.99 in the current study), bringing optimism that other Polynesian-enriched variants may now also be better imputed with this reference panel.

In our current study, we imputed both the Native Hawaiians (max N = 5,376) from the Multiethnic Cohort (MEC) study and Samoans (max N = 3,085) from the Soifua Manuia study using release 3 of the TOPMed imputation reference panel and performed genome-wide meta-analysis for several cardiometabolic traits. Overall, these improvements, coupled with the increased sample size from meta-analyzing Native Hawaiian and Samoan cohorts, allow us to confirm previous suggested associations and also improves power to detect other Polynesian-enriched variants.

## Results

Native Hawaiian and Samoan cohorts were meta-analyzed following association testing. Briefly, genotype array data for each cohort were phased and imputed using the TOPMed imputation server (release 3). After quality control filtering, linear mixed model-based association testing for each of the 13 phenotypes (Table 1, S1-S2 Figs) was performed via GENESIS followed by meta-analysis via METAL. In total, we identified 775 genome-wide significant variants at 25 loci across the autosomes and chromosome X (Fig 1). Quantile-quantile (QQ) plots and the associated lambda did not reveal signs of genome-wide inflation (S3 Fig), nor did we observe significant heterogeneity across cohorts (S1 Table).

**Table 1:** Sample availability for traits analyzed in this study. BMI, body mass index; HDL high-density lipoprotein; HOMA-IR, homeostatic model assessment for insulin resistance; LDL, low-density lipoprotein; T2D, type 2 diabetes. NH T2D: 2564 cases, 2779 controls, Samoan T2D: 483 cases, 2407 controls.

Phenotype	N		
	NH	Samoans	Total
BMI	5,368	3,083	8,451
Fasting Glucose	1,756	2,397	4,153
Fasting Insulin	1,762	2,396	4,158
HDL	1,900	2,858	4,758
Height	5,376	3,085	8,461
Hip Circumference	3,965	3,075	7,040
HOMA-IR	1,725	2,396	4,121
LDL	1,888	2,824	4,712
T2D	5,343	2,890	8,233
Total Cholesterol	1,915	2,859	4,774
Triglycerides	1,896	2,825	4,721
Waist Circumference	3,999	3,074	7,073
Waist-hip ratio	3,939	3,069	7,008

**Fig 1:**
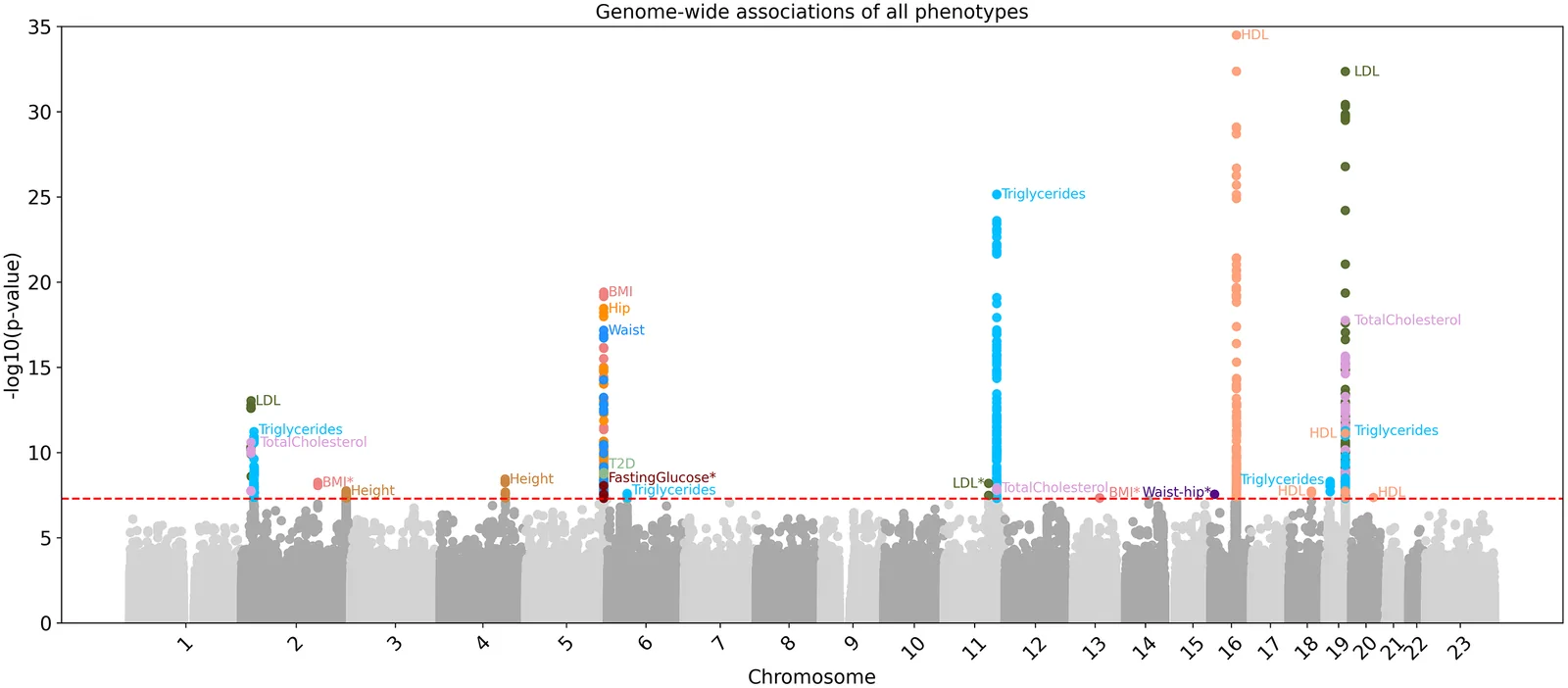
Composite Manhattan plot of meta-analysis associations across all 13 traits. Associations reaching a cutoff of 5e-8 included loci for the following phenotypes: BMI, fasting glucose, HDL, height, hip circumference, LDL, T2D, total cholesterol, triglycerides, waist circumference, and waist-hip ratio. No loci were found to be significant for fasting insulin or HOMA-IR phenotypes.

We classified each phenotype-locus pair as either previously confirmed (replicating a genome-wide significant finding reported in literature or the GWAS catalog [27], which we confirmed through conditional analysis; S2 Table) or putatively new (not previously reported reaching a genome-wide significance threshold of 5e-8). The 25 loci-trait associations were classified as 5 putatively new locus-trait pairs (Fig 2, Table 2) and 20 previously confirmed locus-trait pairs (Figs 3, S4, Table 3). We named each of the 25 loci by the gene with the closest exon at each locus (Tables 2 and 3).

**Table 2:** Summary associations for the loci that are identified as newly confirmed. Lead variant in *CREBRF* was found to be newly genome-wide significantly associated with fasting glucose via meta-analysis. Four additional associations for BMI, LDL, and waist-hip ratio also surpassed genome-wide significance but require additional investigation. The gene with the nearest exon is listed for each variant. NH, Native Hawaiians; AFR, African genetic ancestry group; EAS, East Asian genetic ancestry group; NFE, non-Finnish European genetic ancestry group; AF, allele frequency of the A1 allele; Rsq, imputation r-squared metric for imputation quality. AFR, EAS, and NFE frequency information were retrieved from gnomAD (v3.1.2).

Phenotype	rsid	Chr	Position (hg38)	A1	A2	Meta effect	Meta SE	Meta P-value	Nearest Gene	Region of gene	NH AF	NH Rsq	Samoan AF	Samoan Rsq	AFR AF	EAS AF	NFE AF
**BMI**	rs34964076	2	169265660	c	g	-0.46	0.08	5.7E-09	*LRP2*	Intron	0.015	0.99	0.0017	0.77	0.049	0.0019	0.042
**Fasting Glucose**	rs12513649	5	173045049	g	c	-0.10	0.02	8.9E-09	*CREBRF*	Intergenic	0.090	0.99	0.29	0.99	0.0011	0.068	0.00038
**LDL**	rs142764775	11	98666193	gt	g	15.53	2.68	6.5E-09	*CNTN5*	Intergenic	0.018	0.75	0.023	0.83	0.00005	0.044	0.00003
**BMI**	rs147012253	13	76661594	g	gttc	-1.03	0.19	4.6E-08	*KCTD12*	Intergenic	0.0026	0.92	0.0020	0.57	0.12	0.00000	0.00066
**Waist-hip ratio**	rs138954985	16	7957893	c	g	-0.54	0.10	2.8E-08	*RBFOX1*	Intergenic	0.0019	0.88	0.017	0.95	0.00017	0.0039	0.00000

**Table 3:** Summary associations for the loci that replicate previously reported associations. Among the associations, we replicate known Polynesian-enriched associations with BMI (*CREBRF*), hip circumference (*CREBRF*), waist circumference (*CREBRF*), and HDL (*CETP*). The gene with the nearest exon is listed for each variant. NH, Native Hawaiians; AFR, African genetic ancestry group; EAS, East Asian genetic ancestry group; NFE, non-Finnish European genetic ancestry group; AF, allele frequency of the A1 allele; Rsq, imputation r-squared metric for imputation quality. AFR, EAS, and NFE frequency information were retrieved from gnomAD (v3.1.2).

Phenotype	rsid	Chr	Position (hg38)	A1	A2	Meta effect	Meta SE	Meta P-value	Gene with nearest exon	Region of gene	NH AF	NH Rsq	Samoan AF	Samoan Rsq	AFR AF	EAS AF	NFE AF
Total Cholesterol	rs187411610	2	21167346	g	a	-22.5	3.381	2.6E-11	*TDRD15*	Intergenic	0.0035	0.97	0.019	0.98	0.0000	0.0004	0.0000
LDL	rs187411610	2	21167346	g	a	-23	3.085	9.3E-14	*TDRD15*	Intergenic	0.0036	0.97	0.019	0.98	0.0000	0.0004	0.0000
Triglycerides	rs780093	2	27519736	c	t	-0.07	0.011	6.0E-12	*GCKR*	Intron	0.63	1.00	0.66	0.94	0.8562	0.5075	0.5987
Height	rs7571816	2	232212354	g	a	-0.09	0.016	1.8E-08	*DIS3L2*	Intron	0.30	1.00	0.37	1.00	0.0975	0.5012	0.0222
Height	rs541082621	4	144412450	g	a	0.261	0.044	3.7E-09	*HHIP*	Intron	0.0057	0.97	0.080	0.97	0.0000	0.0023	0.0000
BMI	rs12513649	5	173045049	g	c	0.21	0.023	3.9E-20	*CREBRF*	Intergenic	0.088	0.99	0.28	0.99	0.0011	0.0683	0.0004
Hip circumference	rs12513649	5	173045049	g	c	0.22	0.02	3.6E-19	*CREBRF*	Intergenic	0.086	0.99	0.28	0.99	0.0011	0.0683	0.0004
Waist circumference	rs12513649	5	173045049	g	c	0.21	0.02	6.8E-18	*CREBRF*	Intergenic	0.086	0.99	0.28	0.99	0.0011	0.0683	0.0004
T2D	rs375401688	5	173247705	g	a	-0.39	0.064	1.5E-09	*CREBRF*	Intergenic	0.061	0.95	0.25	0.89	0.0000	0.0006	0.0000
Triglycerides	rs9472143	6	43858616	t	c	-0.067	0.012	2.6E-08	*VEGFA*	Intron	0.23	0.99	0.27	0.88	0.1397	0.1231	0.2394
Total Cholesterol	rs3741298	11	116786845	t	c	-4.593	0.806	1.2E-08	*ZPR1*	Intron	0.51	1.00	0.37	1.00	0.6626	0.5909	0.7970
Triglycerides	rs662799	11	116792991	a	g	-0.114	0.011	7.1E-26	*APOA5*	Upstream variant	0.76	1.00	0.60	0.98	0.8802	0.6990	0.9346
HDL	rs1597000001	16	56971035	t	c	8.97	0.725	3.3E-35	*CETP*	Exon	0.017	1.00	0.035	0.96	0.0000	0.0000	0.0000
HDL	rs117998095	18	50030280	a	g	-5.486	0.976	1.9E-08	*MYO5B*	Intron	0.010	0.96	0.020	0.98	0.0002	0.0363	0.0000
Triglycerides	rs762055973	19	11284678	t	tc	-0.163	0.028	5.0E-09	*TSPAN16*	Intergenic	0.027	0.99	0.040	0.91	0.0000	0.0008	0.0000
HDL	rs429358	19	44908684	c	t	-2.16	0.316	7.5E-12	*APOE*	Exon	0.25	0.99	0.22	0.87	0.2154	0.0975	0.1380
Triglycerides	rs157595	19	44922203	g	a	0.078	0.011	5.3E-12	*APOC1*	Intergenic	0.59	0.95	0.48	0.71	0.8500	0.4278	0.6115
LDL	rs141622900	19	44923535	a	g	-19.7	1.645	4.4E-33	*APOE*	Exon	0.057	1.00	0.065	0.66	0.0814	0.0841	0.0560
Total Cholesterol	rs141622900	19	44923535	a	g	-15.71	1.791	1.8E-18	*APOE*	Exon	0.058	1.00	0.066	0.66	0.0814	0.0841	0.0560
HDL	rs188671999	20	48202897	t	c	21.5	3.923	4.3E-08	*PREX1*	Downstream Variant	0.0012	0.95	0.0012	0.83	0.0003	0.0000	0.0032

**Fig 2:**
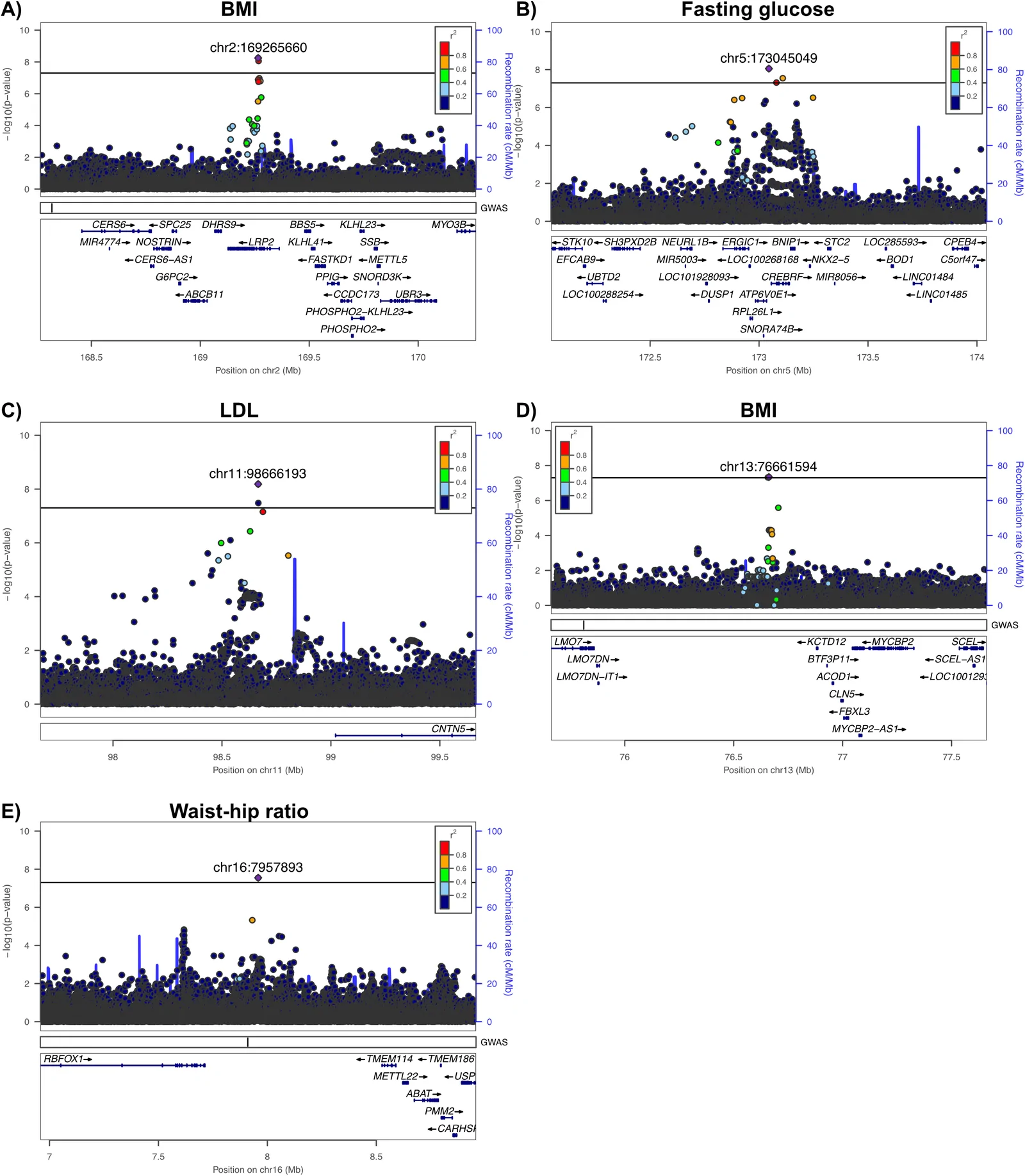
LocusZoom plots for loci categorized as newly confirmed. a) *LRP2* locus for BMI (chr2), b) *CREBRF* locus for fasting glucose (chr5), c) *CNTN5* locus for LDL (chr11), d) *KCTD12* locus for BMI (chr13), e) *RBFOX1* locus for waist-hip ratio (chr16). The GWAS track, if present, shows reported associations in GWAS catalog for the same trait. The recombination rates at each locus reflect inferred rates previously reported for Native Hawaiians in hg38 [28]. LD information is calculated using the NH cohort.

**Fig 3:**
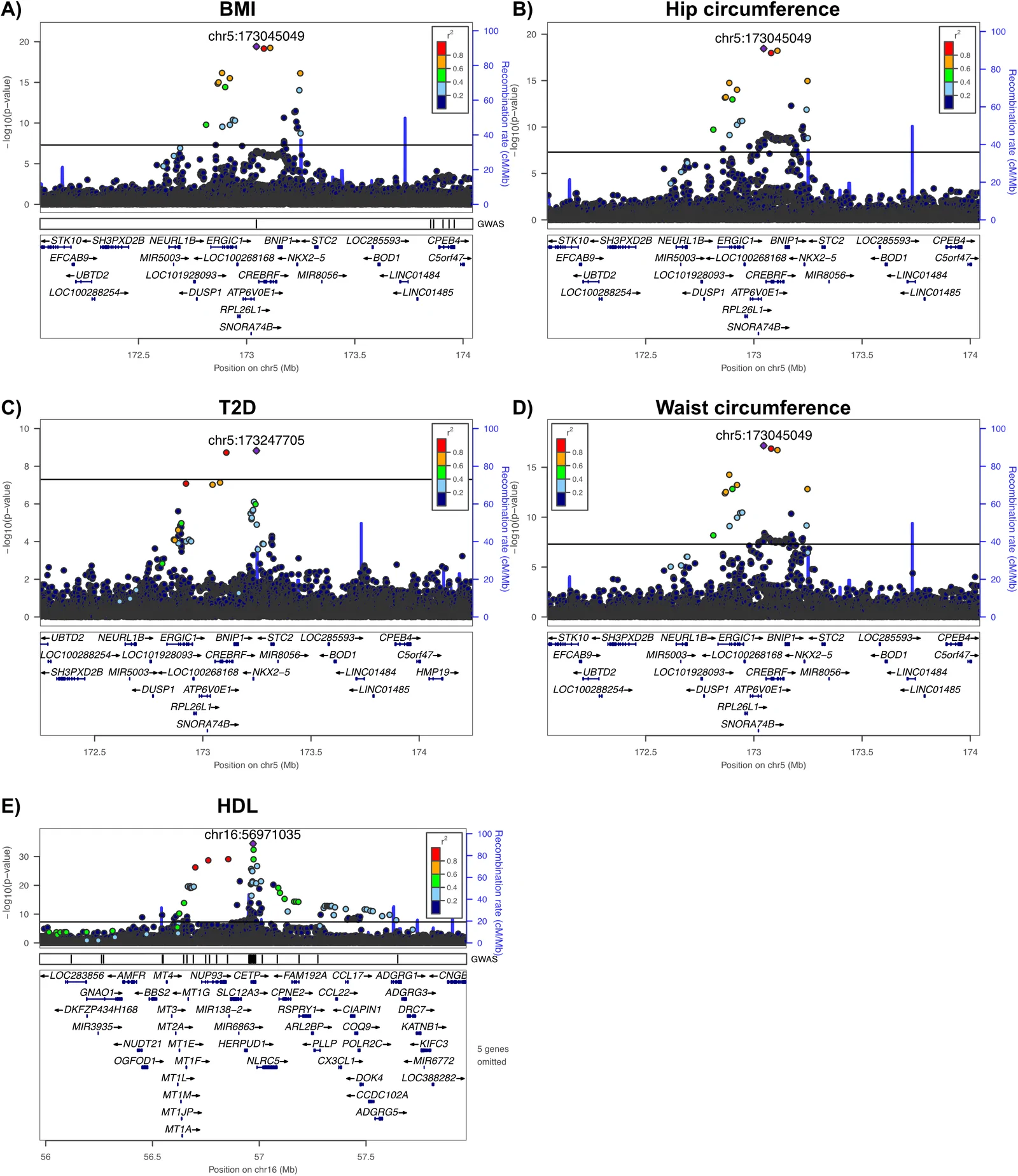
LocusZoom plots for selected loci categorized as previously confirmed. **a)** *CREBRF* locus for BMI (chr5), **b)** *CREBRF* locus for hip circumference (chr5), **c)** *CREBRF* locus for T2D (chr5), **d)** *CREBRF* locus for waist circumference (chr5), **e)** *CETP* locus for HDL (chr16). Remaining replicated loci may be found in S4 Fig. The GWAS track, shown between the plot and gene labels if present, reports associations in GWAS catalog for the same trait. Both genes are known to be Polynesian-enriched. The *CREBRF* missense variant is pleiotropic and its impact on BMI, waist (abdominal) circumference, hip circumference, and T2D have previously been reported. A Polynesian-enriched *CETP* variant has previously been associated with HDL. The recombination rates at each locus reflect inferred rates previously reported for Native Hawaiians in hg38 [28]. LD information is calculated using the NH cohort.

Among the five newly nominated locus-trait pairs, one is located at the Polynesian-enriched *CREBRF* locus and associated with fasting glucose levels. The index variant is not the same missense variant previously reported (rs373863828, chr5:173108771) for association with BMI, though we verified that our top associations reflect the same signal and that there are no additional signals at this locus (S3 Table). The remaining 4 newly confirmed pairs consist of associations for waist-hip ratio (*RBFOX1*, including both sexes), LDL (*CNTN5*), and BMI (*LRP2* and *KCTD12*). For each of these locus-trait pairs, association analysis conditioned on the top associated variant to identify secondary signals found no additional variants reaching genome-wide significance (S3 Table).

A subset of 1,284 Samoan individuals have available WGS data through TOPMed; we found that for the five putatively new associated variants the imputed dosages and genotypes from sequencing are well-correlated (Pearson’s correlation = 0.78-0.99; S4 Table), suggesting that despite the rare to low frequencies estimated for these variants, they were well-imputed for association testing.

Among previously known loci-trait pairs, five loci were associated with triglycerides (*TSPAN16*, *VEGFA*, *APOC1*, *GCKR*, *APOA5*), four with HDL (*MYO5B*, *PREX1*, *CETP*, *APOE*), three with total cholesterol (*TDRD15*, *APOE*, *ZPR1*), two with height (*HHIP*, *DIS3L2*) and with LDL (*TDRD15*, *APOE*), and one with BMI, hip circumference, waist circumference, and T2D (all 4 with *CREBRF*). Each of these loci had at least one reported association within 500 kb of the top variant reported in the GWAS catalog or in literature for the same phenotype. In each case, the observed top association in our study would be strongly attenuated (12 of 20 loci-trait pairs are no longer nominally significant, *P* > 0.05) if we tested an association model including the previously known variant from GWAS catalog (S2 Table), thereby confirming our observed association reflects previously reported associations. The five previously known Polynesian-enriched associations at *CREBRF* (for BMI, waist circumference, hip circumference, T2D) and *CETP* (for HDL) were also identified in the meta-analysis, with no other lead variants at these known loci showing obvious enrichment among Polynesian populations (Table 3). We note that *CETP* locus was previously shown to be associated with HDL and LDL, but in our meta-analysis the association for LDL was marginal (*P* = 0.007238).

We sought to replicate our potentially novel associations using samples in the All-of-Us Research Program (AoU) with 2 analyses [29]. First, because up to 1,221 self-reported Native Hawaiian and Pacific Islander (NHPI) are available in AoU, we tested in AoU all variants with *P* < 1x10^-7^ discovered in our meta-analysis, if the variant is available and if the phenotype is collected. Of the putatively novel association phenotypes, only BMI and waist-hip ratio were available in AoU for the NHPI samples (N = 893) and only matching variants for the BMI loci (on chr2 and 13) were found in the AoU NHPI. In general, we observed no replication at nominal significance (at significance level of *P* = 0.025 for testing two loci; S5 Table), though the association at *KCTD12* was near the significance threshold (*P* = 0.0263).

As no other genetic data from Polynesians are available at scale and accessible to us, we also sought to replicate our putative associations using the multi-ethnic samples in the All-of-Us Research Program (AoU) [29] when the same or related phenotypes were available in a subset of the AoU non-NHPI continental populations. In general, we observed no replication at nominal significance (at significance level of *P* = 0.01, for testing five loci) in any of the variants available (S6 Table). For the associations at *RBFOX1*, *KCTD12*, and *LRP2*, they are relatively common in other continental ancestries (maximum AF in gnomAD AFR, EAS, and NFE 0.0039-0.12). For these loci, we observed no significant association in AoU to waist circumference, BMI (*KCTD12*), and BMI (*LRP2*), respectively, despite having substantial statistical power to replicate (max Power = 90.07%, 100% and 100% in AoU SAS, AFR/AMR/EUR, and AFR/AMR/EUR/SAS, respectively; S6 Table). Combined with the fact that these variants appear to be quite rare in Polynesians (0.0019-0.0180 in NH, 0.0017-0.0168 in Samoans), we concluded that they are likely technical artifacts, possibly due numerical errors in the linear mixed model, or very rare variants that happened to occur in individuals with extreme phenotypes by chance. For associations at the *CREBRF* locus (fasting glucose) and the *CNTN5* locus (LDL), the lead variants or variants in LD in Polynesians were either rare (frequency = 0.00-0.0684) or not tested in sufficiently large AoU populations so we did not have the power to replicate these association using AoU.

Taken together, the association with BMI at *KCTD12* (near nominal significance for replication) and the association with LDL at *CNTN5* (insufficient power to replicate in AoU) loci remain putative associations that await future validation.

## Discussion

In this work, we meta-analyzed Native Hawaiian and Samoan cohorts using imputed dosages from the latest TOPMed imputation server (release 3) for 13 cardiometabolic phenotypes. Despite improved imputation quality and increased sample size compared to previous studies, we do not observe any novel, replicable, commonly occurring Polynesian-enriched variants outside of the previously reported *CREBRF* and *CETP* missense variants.

Our analysis replicated 20 loci previously reported in the GWAS catalog (Table 3). Of the replicated loci, 10 passed the standard GWAS significance threshold of 5e-8 only through meta-analysis. Notably, we are able to detect signals for 5 previously reported Polynesian-enriched associations, *CREBRF* for BMI, hip circumference, waist circumference, and T2D (chr5:173108771, rs373863828) [14,15] as well as *CETP* for HDL (chr16:56971035, rs1597000001) [16]. Despite being Polynesian-enriched alleles, these variants are well imputed with imputation Rsq of 0.991-0.994 and 0.957-1.0 for *CREBRF* and *CETP*, respectively. Though Polynesian-ancestry individuals are still not well represented and thus the reference panel is sub-optimal for imputing the genotypes of Polynesian-ancestry individuals, these two loci underscore the potential of the TOPMed imputation reference for identifying Polynesian-enriched association.

Through increased sample size in meta-analysis, our study was powered to test and confirm the association of *CREBRF* missense variant with fasting glucose (Table 2). This variant had previously been associated with the type 2 diabetes (T2D) phenotype and nominally associated with fasting glucose levels in Samoans, Polynesians, and Pacific Islander cohorts [14,22,30,31]. The remaining 4 novel variant-phenotype associations are relatively rare within our cohorts (AF 0.0017-0.023), with all 4 commonly occurring in at least one global population (MAF > 1%; Table 2). Although we used NHPI from AoU to replicate the putatively new association with BMI on chr2 and 13 (*LRP2* and *KCTD12*), neither locus replicated after accounting for multiple testing. Given the lack of replication, the relatively rare frequency of these variants within our cohort, and the lack of additional evidence in the association of variants in LD to our putative variants, these associations may reflect technical or numerical artifacts. However, this does not rule out that these variants may instead capture the haplotype-tagging of unobserved associations (*e.g., CNTN5* in Fig 2d) or Polynesian-related GxE interactions. In either case, improved genotype imputation or sample size for Polynesian and related populations could help clarify whether these associations are due to an artifact of imputation or unobserved variants.

In our study, meta-analysis of Polynesian cohorts using the latest TOPMed imputation release serves to overcome some of the limitations of previous studies and explore whether more population-enriched associations are present. However, outside of the previously reported *CREBRF* and *CETP* missense variants, we found no additional Polynesian-enriched associations. Given the increased power of our study, our results informed the bounds of potential Polynesian-enriched associations yet to be discovered. It appears clear that both *CREBRF* and *CETP* are unique in terms of the combination of their allele frequency and effect sizes for the traits we examined, and they stand unmatched to other variants we were able to test (S5 Fig). While it is possible that other common Polynesian variants simply do not exert as strong of an effect on these traits, or that the environment has greater contribution to trait variation in these populations, we also note that current state-of-the-art imputation references still have room to improve for underrepresented populations such as the Polynesians and Pacific Islanders. Polynesian-enriched variants that are rare globally are more likely to be absent from the imputation reference or filtered out during the quality control process. Even for variants that can be found in the reference, because of inadequate haplotype representations, Pacific Islander populations are still relatively poorly imputed, particularly for Pacific Islanders with greater Polynesian ancestries compared to those with ancestries better represented in the references [32]. This can be observed for the replicated locus on chromosome 19 around 45 Mb for LDL, triglycerides, and total cholesterol phenotypes: imputation accuracy in the admixed Native Hawaiians individuals (Rsq 0.95-1.00) is higher than the less unadmixed Samoan individuals (Rsq 0.66-0.71). Ergo, a natural future direction would be to use whole-genome sequencing (WGS) data for Polynesian-ancestry individuals to systematically discover Polynesian-enriched variation and create a representative reference panel that accurately imputes the genotype of potential Polynesian-specific variants. A recent study reported that a reference panel with as few as 24 unrelated WGS Samoan individuals could impute as many well-imputed variants (Rsq > 0.8) as the TOPMed panel for a Samoan cohort, and the performance exceeds that of TOPMed when as few as 48 reference individuals were included [33]. This highlights the large impact representation in reference panels may have for diverse populations. Such a reference panel can also be supplemented with other standard reference panels through meta-imputation [34], to reap the benefit of both the breadth of variation coverage across global populations and the depth of population-enriched variation.

In summary, we confirmed a metabolic trait’s association with the *CREBRF* locus in Polynesian populations, further supporting the pleiotropic nature of this locus [14–16]. Although we did not identify other Polynesian-enriched associations for the traits we examined here, we note that there continue to be achievable advancements in available genomic resources, analytical strategies, and cohort aggregation that will allow a more comprehensive investigation to identify important genetic associations, whether for increasing or decreasing risk of complex diseases, or interacting with the environment, in these populations. Identification of these associations will not only reveal new biological insights but also reduce the disparity in how genomic medicine is practiced for these populations, such as the observed disparity in polygenic prediction models [35,36].

## Methods and data

### MEC native Hawaiian cohort

The Multiethnic Cohort (MEC) Study is a prospective epidemiological cohort and consists of over 215,000 individuals from California and Hawai‘i. Height and weight were self-reported upon enrollment for most individuals with follow-up visits to collect biomarker data and questionnaires recording additional phenotypes such as waist and hip circumference. There are over 14,000 Native Hawaiians surveyed in the MEC with genetic data for 5,382 Native Hawaiian individuals available on 2 genotyping arrays, the Illumina multi-ethnic genotyping array (MEGA) and Infinium global diversity array (GDA). There are 4,144 individuals genotyped on MEGA with 1,093,693 variants available and 1,238 individuals genotyped on GDA with 1,549,554 variants available. Number of individuals available for each phenotype can be found in Table 1. Previously described QC protocol was followed prior to GRM inference [15,17,28,37]. Some of the filtering criteria included minimum call rate, minor allele frequency, HWE p-value, poor clustering by visual inspection, mendelian errors, sex difference in allele frequencies for autosomes and sex chromosomes, and positional duplicates.

### Samoan cohort

Participants for the Samoan cohort came from the Soifua Manuia (‘good health’) study, a population-based sample of 3,504 Samoan adults recruited in 2010 [5]. Genotyping was performed for a subset of 3,182 Samoan adults using Genome-Wide Human SNP 6.0 arrays (Affymetrix) at 906,600 positions genome wide and extensive quality control was conducted. Height, weight, abdominal and hip circumferences were measured to the nearest 0.1 mm, 0.1 kg, and 1 mm, respectively, with participants in light island clothing. Measurements of total cholesterol, LDL- and HDL-cholesterol, triglycerides, glucose and insulin were derived from fasting serum samples following standard protocols. Additional details about phenotype measurement, sample genotyping, and genotype quality control have previously been described [5,14].

### Imputation to TOPMed server

Native Hawaiians are available on two array platforms, MEGA and GDA, and each array was independently imputed to the TOPMed imputation server (release 3) to maximize the available variant information for matching to the panel. Samoan array data was also imputed to TOPMed release 3. Before meta-analysis, variants were filtered to have a minimum imputed Rsq of 0.3 and minor allele count (MAC) > 0.

### Phenotype transformation

Previously studied phenotypes (all phenotypes with the exception of hip circumference, HOMA-IR, and waist circumference) followed previous published protocol accordingly (S7 Table) [38]. Hip circumference, HOMA-IR, waist circumference phenotypes were transformed as follows:

HOMA-IR:

fasting insulin (microU/L) x fasting glucose (nmol/L)/22.5

Hip circumference (cm):

sex stratified, regressed against age, and combined residuals

Waist circumference (cm):

sex stratified, regressed against age, and combined residuals

Covariates, transformations, and exclusion criteria were applied to each cohort independently. Individuals were stratified by sex before inverse normal transformations and combined after regressing on sex-specific covariates.

### Association testing with GENESIS

Both cohorts were analyzed using an in-house implementation of a previously reported GENESIS pipeline [39,40]. For Native Hawaiians, inference of PCs and genetic relatedness matrix (GRM) used the intersection of variants between the GDA and MEGA arrays, approximately 1M variants.

This pipeline iteratively infers principal components (PCs) using PC-AiR and infers a GRM using PC-Relate. All programs and software were run with default settings and parameters. To conservatively account for potential structure, we include both the GRM and PCs in association testing when using GENESIS (20 PCs for Native Hawaiian and 3 PCs for Samoan associations).

### Meta-analysis with METAL

METAL [41] was used to meta-analyze the Native Hawaiian and Samoan summary stats using default parameters with the STDERR scheme. Prior to meta-analysis, variants with Rsq < 0.3 were removed from each cohort. Differences in variant ascertainment due to different array platforms may impact the imputation quality for some imputed variants. As a result, additional filtering criteria is added post analysis requiring variants to have imputed Rsq ≥ 0.3 in both cohorts and cross-cohort total MAC ≥ 10 to remove any very poorly or rare imputed associations from meta-analysis. LocusZoom plots use recombination rates reflect inferred rates previously reported for Native Hawaiians in hg38 [28]. We used the conventional threshold of 5x10^-8^ for genome-wide significance. We acknowledge that we did not correct for the multiple correlated traits tested in this study but also note that in previous investigations the testing burden did not always adjust for multiple correlated traits [42,43]. Through principal components analysis of the trait correlation matrix, the additional multiple testing burden would be approximately four to six. We opted to be cautious in interpreting our initial association statistics and sought for external replications in AoU NHPI or other continental populations.

After meta-analysis, the top variants were examined against the GWAS catalog [27] for previously reported variants. Variants within 500kb of the top hit in a region were considered as part of the locus. Categorization using GWAS catalog is determined by the presence of a reported genome-wide significant hit within the locus (P < 5e-8); if such a hit was not present or if the reported hit was not genome-wide significant, the locus is considered newly confirmed. LocusZoom was used to plot the meta-analysis results at each locus with recombination rates (previously reported) and LD calculations (from this study using PLINK 1.90b7.2) for Native Hawaiians [28,44–46].

### Conditional analysis

Conditional analyses were performed at putatively new and previously reported loci. In both cases, imputed dosages for the variant being conditioned on were extracted from the imputed data, included as an additional fixed effect covariate, and association models were re-run for all other variants within 500 kb.

We performed the following conditional analyses based on locus categorization:

1. Putatively new associations
  a. The top signal at each locus was conditioned on to detect potential secondary signals that could be masked by the primary signal.
  b. Phenotypes with associations at the *CREBRF* locus were conditioned on the known Polynesian-specific missense variant (rs373863828) to determine whether these associations may reflect the previously reported variant.
2. Associations with nearby reported or known signal(s)
  a. The known or reported variant in GWAS catalog was conditioned on to verify whether the association from our meta-analysis is reflecting the same signal. In the cases where there was more than one reported signal, conditional analysis used the variant with the most significant association from our meta-analysis rather than the strongest association published in literature.

Quantitative traits have a null model fit with a linear mixed model by GENESIS:


$$\begin{array}{c} y=X\beta +Zb+\epsilon \end{array}$$

where y is the vector of trait outcomes, X is the matrix of fixed effect covariates, β is the vector of fixed effect coefficients, Z is the matrix of random effects, b is the vector of random effects, ϵ is the vector of residual errors. This null model is later used to test variants for associations. In our standard analysis, y is a vector of transformed phenotypes (see **Phenotype transformation**), and X and β have dimensions of N x m and m x 1, respectively, where m is the number of PCs included for each cohort. In our conditional analysis, X and β have dimensions of N x (m + 1) and (m + 1) x 1 to account for the inclusion of the conditioned variant dosage with the PCs. Covariates such as sex, age, BMI, and others are included (depending on trait) during phenotype transformation and therefore not included explicitly during the fitting of the null model.

### All-of-Us replication and power calculation

We attempted to replicate our potentially novel associations using the self-reported Native Hawaiian and Pacific Islander (NHPI) individuals from the All-of-Us Research Program for BMI. For the available 1221 individuals, we transform the available phenotypes to match our meta-analysis while removing individuals with missing covariates. This resulted in BMI as the only phenotype with enough sample size for association after transformation, QC, and intersection with the variants from meta-analysis (N = 893). Association testing was performed using PLINK2 [46] with 20 PCs.

Due to the limited availability of external Polynesian genetic data, we also attempted to replicate our potentially novel associations using the multi-ethnic samples from the All-of-Us Research Program for the same or related phenotypes. Single variant associations for approximately 250,000 individuals were available on the All by All browser (https://allbyall.researchallofus.org). Summary statistics are available for 3,400 phenotypes. The lead variant at each locus was used when available; otherwise, a variant in linkage disequilibrium was used instead. We calculated power for each sub-cohort (AFR, AMR, EAS, EUR, and SAS) using the following R code (significance level for replication, alpha, was set at 0.01):

threshold = qchisq(alpha, df = 1, lower.tail = FALSE)

h2 = 2 * (Effect_meta^2) * af_allele2_aou * (1 - af_allele2_aou)

power = pchisq(threshold, df = 1, lower.tail = FALSE, ncp = N_aou * h2)

where Effect_meta is the effect size from the METAL meta-analysis, af_allele2_aou is the alternative allele frequency, and N_aou is the number of individuals.

### A note on inclusion criteria based on self-reported identity

Throughout this study, we used self-reported identity, as recorded in the Multiethnic Cohort, the Soifua Manuia study, and the All of Us Research Program, to define the study populations included in our analyses. We recognize that self-reported identity and genetically inferred ancestry are distinct constructs. However, using self-reported identity as an eligibility criterion was an explicit study-design choice, made in response to consistent feedback from Native Hawaiian community partners that community membership should be understood through genealogical records and self-identification rather than through quantification of a statistically inferred measure of Polynesian-related ancestry. Applying a quantitative, analyst-defined threshold to include or exclude participants could be divisive to the community. Accordingly, we used self-reported identity to define the study populations, whereas genetic data were used to adjust for potential confounding rather than to adjudicate identity.

## Supporting information

S1 Fig Phenotype distributions for cohorts before unit harmonization and normalization. T2D is the only binary trait of the studied phenotypes. Native Hawaiians (N = 5343): 2564 cases, 2779 controls. Samoans (N = 2890): 483 cases, 2407 controls.
(TIFF)

S2 Fig Miami plot of Native Hawaiian and Samoan cohort associations by phenotype. (TIFF)

S3 Fig Meta-analysis quantile-quantile (QQ) plots by trait. The associated lambdas for each QQ plot were as follows: BMI – 1.04, Fasting Glucose – 1.02, Fasting Insulin – 1.021, HDL – 1.039, Height – 1.083, Hip circumference – 1.02, HOMA-IR – 1.001, LDL – 1.011, T2D – 1.027, Total cholesterol – 1.009, Triglycerides – 1.021, Waist circumference – 1.016, Waist-hip ratio – 1.015.
(TIFF)

S4 Fig LocusZoom plot for remaining loci with known association based on the GWAS catalog. The GWAS track shows reported associations in GWAS catalog for the same trait. The recombination rates at each locus reflect inferred rates previously reported for Native Hawaiians in hg38 [28].
(TIFF)

S5 Fig Statistical power of the current study design. The curves delineate the combination of causal allele frequency and effect sizes (in units of standard deviation, sd, per allele) that could be detected with 80% statistical power in our study assuming a sample size of 8,400 (blue, representing anthropometric traits like BMI) or 4,700 (orange, representing biomarker traits like HDL). For reference, the frequency and effect of the association at the *CREBRF* and *CETP* locus are also shown, colored by their respective trait. Allele frequencies for *CREBRF and CETP* were computed as the weighted average of the Native Hawaiian and Samoan data. It appears that no other common Polynesian-enriched variants exert nearly as large of an effect or segregate at as high of frequency as these two previously found associations.
(TIFF)

S1 Table Heterogeneity metrics from meta-analysis. METAL was run with “ANALYZE HETEROGENEITY” and the output for all 25 top hits at each loci-variant pair are provided. HEtISq, HetChiSq, HetDf, and HetPVal reflect I^2^ statistic, chi-squared statistic, degrees of freedom, and P-value respectively.
(XLSX)

S2 Table Conditional analysis at loci with previously known associations. Conditional analyses were conducted using the dosage of the previously reported variant in the GWAS catalog. If multiple nearby hits were found, the variant with the strongest association in our meta-analysis was used. Across all loci, association signals decreased drastically after conditional analysis leading us to conclude that the initial signals reflect previously reported findings.
(XLSX)

S3 Table Conditional analysis in newly confirmed loci. Two separate conditional analyses were performed for the loci with potentially novel associations. First, in order to detect potential secondary signals, we conditioned on the lead variant at each locus and report the smallest p-value within 500kb. No additional signals were detected from this analysis. Second, in order to determine if the signal at the CREBRF locus is in fact reflecting the known missense variant, we conditioned on rs373863828 (chr5:173108771:C:G). We find that the signal at the locus decreases significantly and conclude that the original signal is due to the CREBRF missense variant.
(XLSX)

S4 Table Pearson correlation of imputed dosages and sequenced genotypes at putatively novel loci. WGS data is available for 1284 Samoan individuals. Pearson correlation of the TOPMed imputed dosages at our nominated loci with the sequenced genotype on available individuals show they are well correlated.
(XLSX)

S5 Table Replication results at implicated loci from meta-analysis using 893 Native Hawaiian and Pacific Islander (NHPI) samples in All-of-Us (AoU). We attempt to replicate our findings for available phenotypes in AoU. After transformation and QC, BMI and waist-hip ratio were the only phenotypes available for replication and BMI the only phenotype with the putatively novel variants. We tested in AoU all variants with *P* < 1x10^-7^ in the meta-analysis in the two loci associated with BMI (chr2 and chr13). Neither locus revealed nominally associated variants with BMI after adjusting for multiple testing (*P*-value threshold = 0.025). AOU_A1 is the effect allele.
(XLSX)

S6 Table Power to replicate potentially novel variant-trait association in All-of-Us (AoU). We calculated power to replicate a finding in AoU assuming effect sizes in units of standard deviation from our meta-analysis but using frequency and sample size of the AoU cohort for replication. The critical value threshold used in power calculation was 6.6349, which was calculated for P = 0.01. For fasting glucose and LDL the units of the associated results were converted into units of standard deviation. Most of the phenotypes from the novel loci-phenotype pairs are available in AoU, with the exception of waist-to-hip ratio; for waist-hip ratio, we use mean waist circumference, a related phenotype for replication. As previously reported, a CREBRF variant (rs12513649) is found more commonly across global populations in AoU, while the causal missense variant (rs373863828) is extremely rare. Rs12513649 is in LD with rs373863828 among Polynesians and thus was the top associated variant in our study. However, despite being powered to detect associations with rs12513649 in AMR and EAS populations, it is not replicated in AoU likely because the variant is not in LD with the causal variants outside of Polynesia. Rs373863828 was not tested by AoU due to its extreme rarity. Lastly, for the AFR subcohort in AoU, associations with rs12513649 are testing the T alternative allele rather than the G allele, which is not available. AFR – African ancestry, AMR – Admixed American, EAS – East Asian, SAS – South Asian, EUR – European.
(XLSX)

S7 Table Criteria, units, and covariates for phenotype transformation are listed for tested quantitative traits. The number of PCs included in the linear mixed model, in addition to a GRM, is 20 for the Native Hawaiian cohort and 3 for the Samoan cohort. INT – inverse normal transformed. Following the practice in PAGE consortium [38], for lipids in Native Hawaiians we adjusted for medication use with the following heuristics: HDL (mg/dL; statins: -2.3; fibrates: -5.9; bile acid sequestrants: -1.9; niacin: -9.9, 247 cholesterol absorption inhibitors: + 0.0; LDL (mg/dL; statins: + 49.9; fibrates: + 40.1; bile acid sequestrants: + 40.5; niacin: + 24.7; cholesterol absorption inhibitors: + 40.5; Total cholesterol (mg/dL; statins: + 52.1; fibrates: + 46.1; bile acid sequestrants: + 0.0; 300 niacin: + 34.6; cholesterol absorption inhibitors: + 40.5; Triglycerides (mg/dL; statins: + 18.4; fibrates: + 57.1; bile acid sequestrants: + 0.0; 319 niacin: + 89.4; cholesterol absorption inhibitors: + 0.0. For Samoans we do not have detailed medication information, but individuals are excluded prior to enrollment if they reported use of heart disease medication.
(XLSX)
